# Single‐Step Breeding Value Estimations and Optimum Contribution Selection in Endangered Dual‐Purpose *German Black Pied Cattle* (DSN) Using a Breed Specific SNP Chip

**DOI:** 10.1111/jbg.12929

**Published:** 2025-02-01

**Authors:** M. Wolf, T. Yin, G. B. Neumann, P. Kokuć, G. A. Brockmann, S. König

**Affiliations:** ^1^ Institute of Animal Breeding and Genetics Justus‐Liebig‐University Gießen Gießen Germany; ^2^ Animal Breeding Biology and Molecular Genetics, Albrecht Daniel Thaer‐Institute for Agricultural and Horticultural Sciences Humboldt Universität zu Berlin Berlin Germany; ^3^ Leibnitz Institute for Zoo and Wildlife Research Berlin Germany

**Keywords:** local cattle, optimum genetic contributions, single‐step genetic evaluation

## Abstract

The aims of the present study were to perform single‐step genomic predictions in the dual‐purpose *German Black Pied cattle* (DSN) breed considering a DSN specific SNP chip (DSN_200 K), and to use the corresponding estimated breeding values (EBV) in ongoing optimum genetic contribution (OGC) selection. All results were compared with the application of the commercial *Illumina BovineSNP50 BeadChip* (50 K). The traits of interest in the present study (due to the differing breeding history of these traits in the past) included 305‐day lactation protein percentage (Pro%) of 9029 DSN cows, fat‐to‐protein ratio (FPR) from the first test‐day of 8773 DSN cows, and stature (STAT) measured in cm of 4409 DSN cows. The DSN cows represented the calving years 2008–2019. Genotyping of 2797 DSN animals was conducted using both the DSN_200 K and the 50 K. From the genotyped animals, a subset of 1800 cows had phenotypic records for all three traits FPR, Pro% and STAT. Heritabilities from the single‐step genetic parameter estimations were quite large for Pro% (0.69) and STAT (0.78), but small for FPR (0.11). The choice of the SNP chip only had minor effects on variance components, heritabilities and EBVs. Furthermore, genetic parameters were very similar from genetic‐statistical models additionally considering a linear regression on pedigree‐based inbreeding coefficients. OGC selection was applied to a pool of 1125 pre‐selected bull sires (BS) and bull dams (BD). A more relaxed genetic relationship constraint was associated with favourable effects on the average EBVs for Pro%, FPR and STAT, and a declining number of selected BS. The gains in genetic merit were marginal when relaxing the constraint at 0.06 for the genetic relationships or higher. The same associations were found for an overall breeding index (I‐DSN), considering the three traits with equal weights. Consequently, we suggested OGC applications with a genetic relationship constraint of 0.06, which contributed to genetic gain in I‐DSN of 17.9%, and to increased diversity due to an increased number of BS, when compared to the current practical elite animal selection scheme. A large number of finally selected BS and BD was identical when either using EBV from the DSN_200 K or from the 50 K. From such perspective, we only see marginal extra value for the specific DSN SNP‐chip application.

## 1 Introduction

Local dual‐purpose *German Black Pied cattle* (DSN, German: Deutsches Schwarzbuntes Niederungsrind) with a population size of ~2500 cows is the founder breed of modern Holstein Friesian (HF) in Germany (Jaeger, Brügemann, Naderi, Brandt, and König, 2018; Jaeger, Scheper, König, and Brügemann, 2018). Due to the breeding history and still existing genetic relationships with HF (Naderi et al. 2020), official genetic evaluations of DSN are embedded in the infrastructure as developed for HF. Nevertheless, divergent breeding strategies in DSN and HF over the past decades with focus on different trait categories contributed to a genetic differentiation between both selection lines. The genetic differentiation resulted in linkage disequilibrium pattern (Naderi et al. 2020), in allele frequencies for important genes such as *DGAT1* (Korkuć et al. 2023), and in allele substitution and dominance effects for selected SNP (May et al. 2019), which differed in the HF and the DSN population.

The breeding goal definition for DSN during the past decades consistently emphasised lactation protein percentage (Pro%). A small body size reflected via measurements in cm for stature (STAT) was a breeding objective from the late 1970s until 2015, but in commercial farms, intra‐herd selection strategies recently favoured taller cows. The idea behind a breeding focus on small STAT was to be complementary with the conformation characteristics of modern HF cows, and to achieve low body weights, that is, following the cattle breeding approach as implemented for pasture based systems in New Zealand (Alemu, Handcock, and Garrick 2023). Breeding on small‐sized cattle might favour energy efficiency aspects and grassland suitability, but for the dual‐purpose DSN, also carcass traits and body weight play an economic role (Meier et al. 2021). Consequently, current DSN breeding efforts favour an increase of body measurements and weights. The fat‐to‐protein ratio (FPR) in early lactation has been suggested as a reliable indicator for metabolic stability (Jaeger, Brügemann, Naderi, Brandt, and König, 2018; Jaeger, Scheper, König, and Brügemann, 2018; Klein et al. 2019). Furthermore, Bergk and Swalve (2011) identified strong associations between FPR from test‐days in early lactations with cow longevity. Consequently, practical DSN breeding aims on the consideration of FPR, but the intermediate trait optimum (a low FPR indicates acidosis, a high FPR indicates ketosis), implies some difficulties in this regard. Overall, different selection strategies have been applied for Pro%, STAT and FPR in the DSN breed in the past. Hence, these traits are interesting for investigations addressing selection and mating designs.

The commercial *Illumina BovineSNP50 BeadChip* (hereafter referred to as 50 K) as designed for the large dairy populations does not fully reflect the genetic characteristics and the importance of specific traits in DSN. Consequently, Neumann et al. (2021) developed a breed specific *DSN 200 K SNP chip* (DSN_200 K), which especially considered variants and genomic regions with major effects on Pro%, STAT and FPR (May et al. 2022). The DSN_200 K was used for the estimation of genetic parameters for production and conformation traits based on the genomic relationship matrix (Wolf et al. 2023), and for genome wide association studies (Korkuć et al. 2023). Estimated heritabilities were slightly larger and association signals were clearer compared to 50 K SNP chip applications. Not all DSN cows are genotyped, but trait recording comprises the whole population. Hence, accuracies of genomic predictions might increase when combining genomic and pedigree‐based approaches in single‐step genetic evaluations. Such effects were clearly elaborated for genetic evaluations in organic populations with scarcely genotyped cows (Shabalina et al. 2020). The methodology to combine pedigree and genomic relationships through the so‐called H‐matrix is described, by, for example, Legarra et al. (2014).

An issue following the genetic evaluations addresses the selection and mating scheme. Especially for small‐sized populations, it is imperative to monitor and to manage inbreeding and genetic relationships in a long‐term perspective. In the DSN population, selection for females and natural service sires is mostly based on phenotype characteristics, and on EBVs for the three major traits for cow and bull sires (Jaeger et al. 2018a). Some large‐scale DSN herds utilise mating software for the selection of cow sires for intra‐herd replacements, but already existing close relationships among sires from artificial insemination (AI) programs narrow the availability of alternative or outcross genetics. Based on historical datasets spanning a 40 years period from 1975 to 2005, trait responses on inbreeding alterations were quite stable, disproving any significant inbreeding depressions for primary as well as for functional traits (Jaeger et al. 2018a). Nevertheless, high relationships of some recent influential sires with the current cow population point to detrimental inbreeding effects in the near future. Also in an international study simultaneously considering DSN populations from Germany, Poland and The Netherlands, high genetic relationships among the most popular bulls used for AI, were identified (Jaeger et al. 2018b). Close genetic relationships among influential sires imply a time‐lagged increase of genetic connectedness, a loss of genetic diversity and associated possible future inbreeding depressions in the DSN cow population. Hence, it is imperative that selection strategies aiming on the conservation of genetic variability in the long‐term perspective regulate genetic relationships among bull sires and bull dams. In this regard, the optimum genetic contribution (OGC) concept as developed by Meuwissen (1997) successfully has been applied in dairy cattle breeding schemes, that is, to determine optimal mating frequencies for bull sires and bull dams (König and Simianer 2006).

Following all the constraints, challenges and background information as stated above, the aims of the present study were (a) to perform single‐step genetic evaluations for DSN on the basis of the newly developed DSN_200 K for the traits FPR, Pro% and STAT, and (b) to use the estimated breeding values (EBV) and an overall index for an OGC approach to select DSN bull sires and bull dams. Genetic parameters from the single‐step genomic predictions, and genetic merit and the number of selected bull sires from the different OGC scenarios, were compared with commercial 50 K chip applications.

## 2 Materials and Methods

### 2.1 Phenotypes

Phenotypic records of first parity DSN cows comprised the calving years 2008–2019. In this study, we used FPR from the first official test‐day after calving from 9029 cows, 305‐day lactation records for Pro% from 8373 cows, and STAT measurements from 4409 cows. For FPR, previous data editing excluded first test‐days outside the period between 5 and 40 days after calving. Only completed 305‐day lactations were considered for Pro%. In a subset of the eight largest herds, STAT in cm was measured in the process of official type trait classification. The descriptive trait statistics are given in Table 1. The DSN cows recorded for FPR and Pro% were kept in 13 herds, with an average herd size of 63 cows and a minimum of 23 cows per herd and a maximum of 473 cows per herd. However, apart from two large‐scale farms located in former East Germany (average herd size: 378 cows), the DSN cows were mostly kept in small‐sized family farms with focus on organic or grazing systems.

**TABLE 1:** Descriptive statistics for the cow traits 305‐day lactation protein percentage (Prot%), fat‐to‐protein ratio (FPR) at the first test‐day and stature (STAT).

Trait	Mean	SD	Min.	Max.
Pro% (in %)	3.48	0.24	2.45	4.31
FPR (a ratio)	1.22	0.21	0.41	3.03
STAT (in cm)	135.38	5.61	125.00	143.00

### 2.2 SNP Chips and Genotyping

Genotyping considered 2797 DSN animals. From the genotyped animals, a subset of 1800 cows had phenotypic records for all three traits FPR, Pro% and STAT. Further 997 genotyped animals were sires, paternal or maternal grandsires of the cows with phenotypes. Genotyping was performed using two different SNP chips. First, the standard 50 K chip with ~54,000 markers as commonly used for genetic evaluations of the commercial breeds HF and Simmental, was used. The second SNP chip was the DSN specific DSN_200 K. Our approach in developing the DSN_200 K based on whole‐genome sequence data with in total 20,587,181 sequence variants is outlined by Neumann et al. (2021). For SNP quality control for the genotyped DSN cattle in the present study, we used the software package PLINK (Purcell et al. 2007). Filtering of SNP genotypes comprised the standard criteria, that is, exclusion of SNPs with a call rate smaller than 0.01, a minor allele frequency smaller than 0.05 and significant deviation (*p* < 1 × 10^−8^) from Hardy–Weinberg equilibrium, and locations on a sex chromosome. After the filtering steps, 31,248 SNPs (50 K chip) and 152,493 SNPs (DSN_200 K chip) from the 2797 animals, were available for the ongoing genomic analyses.

### 2.3 Inbreeding and Genetic Structure

Pedigrees were corrected and some gaps were filled using our own algorithm specifically developed for the DSN breed (Jaeger, Brügemann, Naderi, Brandt, and König, 2018; Jaeger, Scheper, König, and Brügemann, 2018). The DSN pedigree (only considering DSN animals with a DSN breed percentage larger than 90%) comprised in total 19,593 animals, traced back to the oldest founder animal born in 1906. The second oldest animal in the dataset was born in 1922. All birth years from 1952 to 2019 were represented in the pedigree.

For the genotyped animals, we computed the pedigree‐based relationship matrix A using the CFC computer package (Sargolzaei, Iwaisaki, and Colleau 2006). A pedigree‐based inbreeding coefficient larger than 0 was calculated for 12,203 animals. The average inbreeding coefficient considering all animals from birth years 1952–2019 was 0.013, with a current value of 0.038 for animals born in 2019. For the cows with phenotypic records, the average inbreeding coefficient was 0.034. The average generation interval on the cow‐dam pathway of selection for the cows with phenotypes comprised 5.9 years. Criteria reflecting the depth of the pedigree were calculated using CFC. The values for the longest ancestral path and the average number of discrete generation equivalents were 22 and 6.45, respectively. The pedigree completeness index according to MacCluer et al. (1983) for the cows with phenotypic records was larger than 96% for all birth years.

### 2.4 Single‐Step Genetic Evaluations

Genetic parameters and breeding values for FPR, Pro% and STAT were estimated applying single‐step GBLUP methodology, as implemented in the packages PREGSF90 and POSTGSF90 from the BLUPF90 program family (Aguilar et al. 2014). In this regard, we defined the following single‐trait animal Model 1:

(1) y=Xβ+Za+e

where *y* is a vector of observations for FPR, Pro% or STAT; *β* is a vector of fixed effects including herd, calving year, calving season (spring, summer, autumn, winter) and age at first calving for FPR and Pro%, and additionally the classifier for STAT; a is a vector of random additive genetic effects, with *a* ~ N(0, H σ^2^_a_), and *σ* ^2^_a_ denoting the additive genetic variance and H denoting the combined (pedigree and genomics) relationship matrix constructed according to Legarra, Aguilar, and Misztal (2009); *e* is a vector of random residual effects with *e* ~ *N*(0, Iσ^2^_e_) with *σ* ^2^_e_ denoting the residual variance; and *X* and *Z* are the incidence matrices for fixed and additive genetic effects, respectively. The single‐step genetic parameter estimations were conducted in consecutive runs based on the 50 K and the DSN_200 K genotypes using the same pedigree, the same data, and the same base populations. We run two sub‐models in this regard, with or without a linear regression on the pedigree‐based inbreeding coefficient.

### 2.5 Optimum Genetic Contribution Selection

The OGC application aims to maximise genetic gain, assuming that higher EBVs are also favourable in the sense of breeding, which implies challenges for traits with an intermediate optimum. Consequently, for FPR, we transformed the original EBVs to reflect the physiological and economic background in ongoing OGC applications. A high original FPR EBV indicates ketosis, and a low original FPR EBV indicates acidosis. According to Bergk and Swalve (2011), an intermediate original EBV for FPR of exactly 0 or an average phenotypic value of 1.1 was related with smallest frequencies for involuntary cow disposals. Consequently, for all animals, the difference of the maximal EBV for FPR (1.05 from the 50 K genetic evaluation, 1.09 for the DSN_200K genetic evaluation) minus the absolute value of the original EBV for FPR, was calculated. Such procedure implied highest transformed FPR EBVs for animals with original EBV of 0 (= favourable in the sense of breeding), and smallest transformed FPR (transformed EBV = 0) for extreme animals.

For the OGC applications, we additionally constructed an overall index (I‐DSN), considering the three EBVs for FPR, Pro% and STAT with equal weights (33.3% each). The I‐DSN was standardised to a mean of 100 and a SD of 12 points.

The OGC approach focused on the selection of elite animals, that is, bull sires (BS) and bull dams (BD), for a genomic breeding program as outlined by Schaeffer (2006). In this regard, we aimed on determining the optimal genetic contributions of BS and BD to generate the next cohort of young bulls (YB). The pool of possible selection candidates as pre‐selected by the DSN breeding organisation for genotyping comprised 1125 active animals (48 genotyped BS and 1077 genotyped BD from the 13 herds). The generated YB are used for AI and/or as natural service sires in a rotational system, implying that natural service sires are exchanged across herds in distinct intervals. Such strategy in utilising natural service sires enables gene flow from specific sires in all DSN herds. The DSN breeding organisation generates 15 YB per year. The guidelines of organic farming in Germany strongly restrict the utilisation of reproduction biotechnologies, especially of embryo transfer. Hence, the application of selection schemes without embryo transfer and semen sexing requires the selection of 30 BD to produce 15 YB. Theoretically, in an AI program, one specific outstanding BS could be mated with all 30 BD. Nevertheless, we defined a maximal constraint in this regard, that is, fixing the maximal genetic contribution of a male selection candidate to 0.02 = 20%. The minimal genetic contribution of an individual selected BS is a single mating with one BD, that is, 1/30 = 0.033 = 3.3%.

The applied OGC algorithm to determine optimal mating frequencies (= genetic contributions) for BS and BD followed the equations as developed by Meuwissen and Sonesson (1998), and as implemented in the respective software package GENCONT. Input parameters were the single‐step EBVs for FPR, Pro% and STAT, and I‐DSN of the 1125 potential selection candidates (stored in vector u in consecutive runs for the different traits) and the respective sex information. The genetic contributions of the selected elite animals are included in vector c. In brief, the maximisation of selection response in each single trait implies to maximise c΄u, by constraining the average genetic relationships through c'Ac in the range from 0.02 to 0.10 in consecutive runs. Major output criteria were the average EBV of selected elite animals, and the number of selected BS at the constraint for genetic relationships.

## 3 Results

### 3.1 Single‐Step Genetic Parameters and Breeding Values

The variance components and the respective heritabilities for FPR, Pro% and STAT are given in Table 2. For the two different SNP chips (50 K or DSN_200 K) and from the two different sub‐models (with or without a linear regression on the pedigree‐based inbreeding coefficient), the genetic variances and residual variances were very similar for the same trait, implying heritabilities in the narrow range from 0.691 to 0.697 for Pro%, from 0.106 to 0.108 for FPR, and from 0.777 to 0.780 for STAT. Consideration of the pedigree‐based inbreeding coefficient in the statistical model had no effect on variance components and heritabilities. All heritabilities were associated with small SE in the range from 0.016 (FPR) to 0.022 (Pro%).

**TABLE 2:** Single‐step variance components and heritabilities with respective SE for 305‐day protein percentage (Pro%), first test‐day fat‐to‐protein ratio (FPR) and stature (STAT) based on genotypes from the commercial 50 K Chip (50 K) and the specific DSN chip (DSN_200K) from Model 1 with and without linear regression on pedigree‐based inbreeding.

Trait	SNP chip	Model	Genetic variance	Residual variance	h^2^	SE
Pro%	50 K	Without inbreeding	0.0276	0.1233	0.6912	0.022
		With inbreeding	0.0276	0.0123	0.6913	0.022
	DSN_200 K	Without inbreeding	0.0279	0.0121	0.6967	0.022
		With inbreeding	0.0278	0.0121	0.6968	0.022
FPR	50 K	Without inbreeding	0.2608	2.1915	0.1061	0.020
		With inbreeding	0.2612	2.1914	0.1062	0.016
	DSN_200 K	Without inbreeding	0.2659	2.1871	0.1081	0.020
		With inbreeding	0.2663	2.1871	0.1083	0.020
STAT	50 K	Without inbreeding	9.6249	2.7515	0.7771	0.017
		With inbreeding	9.6755	2.7563	0.7777	0.017
	DSN_200 K	Without inbreeding	9.6626	2.7106	0.7803	0.017
		With inbreeding	9.5528	2.7123	0.7788	0.017

For the same trait but different chip applications (50 K vs. DSN_200 K), the mean, minimum and maximum EBVs as well as I‐DSN were very similar (Table 3) for the whole dataset (including the cows with phenotypic records as specified in Table 1 and their sires), as well as for the group including the 1125 pre‐selected animals for OGC applications. Interestingly, the selection candidates had lower EBV for STAT compared to all animals, reflecting the selection strategy on small‐sized DSN cows in the breeding herds, but on larger animals in commercial herds.

**TABLE 3:** Descriptive statistics for estimated breeding values for protein percentage (Prot%), fat‐to‐protein ratio (FPR), transformed FPR (FPR‐Trans)^a^, stature (STAT) and the overall index (I‐DSN)^a^ for all animals (ALL; including the cows with phenotypic record as specified in Table 1 and their sires), and for the 1125 pre‐selected elite animals for optimum genetic contribution selection (ELITE) from the different SNP chips 50 K and DSN_200 K.

Trait	Group	Chip	Mean	SD	Min.	Max.
Pro% (in %)	ALL	50 K	0.029	0.125	−0.479	0.604
	ALL	DSN_200 K	0.028	0.126	−0.483	0.609
	ELITE	50 K	0.138	0.131	0.320	0.686
	ELITE	DSN_200 K	0.140	0.130	0.339	0.689
FPR (a ratio)	ALL	50 K	−0.027	0.212	−1.073	1.052
	ALL	DSN_200 K	−0.029	0.213	−1.058	1.087
	ELITE	50 K	−0.137	0.262	−1.073	0.878
	ELITE	DSN_200 K	−0.133	0.263	−1.058	0.864
FPR‐Trans	ALL	50 K	0.355	0.115	0	1.052
	ALL	DSN_200 K	0.360	0.120	0	1.087
	ELITE	50 K	0.242	0.154	0	0.878
	ELITE	DSN_200 K	0.248	0.157	0	0.864
STAT (in cm)	ALL	50 K	−4.847	5.386	−18.313	11.576
	ALL	DSN_200 K	−4.829	5.379	−18.309	11.642
	ELITE	50 K	−9.662	2.017	−15.762	−0.325
	ELITE	DSN_200 K	−9.631	2.026	−15.723	−0.355
I‐DSN	ALL	50 K	101.48	11.01	74	136
	ALL	DSN_200 K	101.93	11.43	72	137
	ELITE	50 K	105.80	9.49	96	152
	ELITE	DSN_200 K	106.02	9.64	96	153

^a^ Transformations of EBV and calculations of the index are explained in the materials and methods.

### 3.2 Inbreeding Effects and Optimum Genetic Contribution Selection

From Model 1 additionally considering a linear regression on the pedigree‐based inbreeding coefficient, respective results depict the change in the unit of the trait per 0.01 increase in inbreeding (Table 4). The effects of pedigree inbreeding coefficients on Pro%, FPR and STAT were quite small. For Pro%, 0.01 pedigree‐based inbreeding increase was associated with a reduction of 0.003% (50 K) and 0.002% (DSN_200 K) in milk protein content. Increased inbreeding was associated with smaller body sizes, that is, a reduction of 0.062 cm per 0.01 pedigree inbreeding coefficient (50 K), and of 0.059 cm per 0.01 pedigree inbreeding coefficient (DSN_200 K). The effects on FPR were negligible with regression coefficients very close to zero. However, for FPR, as outlined before, smallest and highest values reflecting acidosis and ketosis, respectively, are unfavourable. Consequently, a linear regression on inbreeding cannot fully depict the underlying physiological mechanisms. Consequently, in pure phenotypic association analyses, we classified the cows according to FPR as follows: (i) FPR < 1.0 indicating acidosis, (ii) FPR > 1.6 indicating ketosis, and (iii) the intermediate class with FPR between ≥ 1.0 and ≤ 1.6. The average pedigree‐based inbreeding coefficient was smallest for the intermediate cow group (0.031), but higher in the acidosis (0.0350) and ketosis group (0.034).

**TABLE 4:** Regression coefficients with respective standard errors (SE) and test for significance^a^ for the change in protein percentage (Prot%), fat‐to‐protein ratio (FPR) and stature (STAT) per 0.01 increase of the inbreeding coefficient from the genetic‐statistical model using the 50 K or the DSN‐200 K genotype data.

Trait	Chip	Regression coefficient	SE	Significance
Pro% (in %)	50 K	−0.003	0.001	n.s
	DSN_200 K	−0.002	0.001	n.s.
FPR (a ratio)	50 K	0.001	< 0.000	n.s.
	DSN_200 K	0.001	< 0.000	n.s.
STAT (in cm)	50 K	−0.062	0.008	n.s.
	DSN_200 K	−0.059	0.008	n.s.

^a^ *Note:* n.s.: Regression coefficient is not significantly different from zero (*p > 0.05*).

**FIGURE 1:**
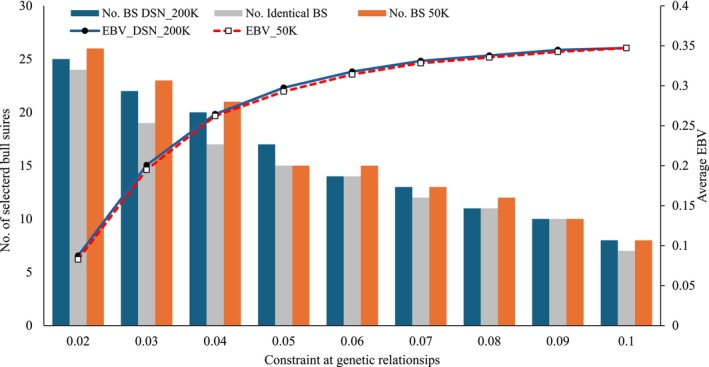
Average estimated breeding values (EBV) for protein percentage of selected bull sires (BS) and bull dams and the number of selected BS at constraint for average genetic relationships. (DSN_200 K = the DSN specific SNP chip was used for the genetic evaluation; 50 K = the standard 50 K chip was used for the genetic evaluations). [Colour figure can be viewed at wileyonlinelibrary.com]

Figure 1 displays the main output parameters for the OGC selection for Pro%, that is, the average genetic merit of the selected parents and the number of selected BS at different constraint for genetic relationships. Genetic gain in terms of average EBVs for Pro% is increasing with a relaxing constraint in genetic relationships. The average EBV of selected animals was 0.083 (50 K) and 0.087 (DSN_200 K) at a strict constraint of 0.02 for the genetic relationships, but 0.347 at a relaxed genetic relationship constraint of 0.10. However, the increase in genetic merit per additional 0.01 relaxed relationship constraint was larger at strict restriction levels compared to already relaxed restriction levels. At an already relaxed constraint of 0.07 genetic relationships, further relaxations were associated with only minor positive effects on genetic merit. As expected, a strict constraint in genetic relationships was related with more diversity in terms of selected BS, that is, an increased number of different BS. For the strict constraint at 0.02 genetic relationships, 25 (DSN_200 K) and 26 (50 K) BS were selected, but at a 0.10 genetic relationship constraint, only 8 influential BS heavily contributed with gene flow to the next generation. At most of the genetic relationship constraint, the slightly higher genetic merit based on the DSN_200 K EBV was associated with a slightly lower number of BS compared to the selection based on the 50 K EBV. The effects of the genetic relationship constraint on the number of selected BS were very similar based on the “50 K EBV” or on the “DSN_200 K EBV”. The similarity is reflected through the large overlap of same BS which have been selected in both scenarios. For example, at 0.10 genetic relationship constraint, from the 8 selected BS, 7 BS were identical.

**FIGURE 2:**
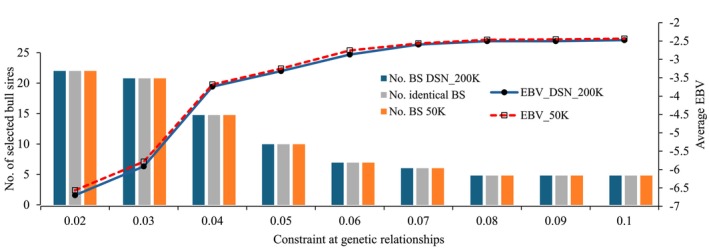
Average estimated breeding values (EBV) for stature of selected bull sires (BS) and bull dams and the number of selected BS at constraint for average genetic relationships. (DSN_200 K = the DSN specific SNP chip was used for the genetic evaluation; 50 K = the standard 50 K chip was used for the genetic evaluations). [Colour figure can be viewed at wileyonlinelibrary.com]

Figure 2 displays genetic merit and the number of selected BS at different constraint for genetic relationships for the OGC selection for STAT. The current selection change towards larger cows implies that higher STAT EBV are favourable. Consequently, a more relaxed constraint for genetic relationships was associated with an increase of the average EBV for STAT, due to a lower number of selected BS with favourable (higher) STAT EBVs. However, at all genetic relationship constraints, the average EBV were negative, indicating the generally low STAT EBV among the current pool of selection candidates, and the above mentioned strategy on small‐sized cattle in the past two decades. Nevertheless, the strict OGC application contributed to the desired breeding objective with focus on a larger body size towards the population average. Again, the increase in genetic merit was very obvious when relaxing the constraint at a generally low level for genetic relationships. The effect on genetic gain based on EBVs from either the 50 K or the DSN_200 K was very similar, because the breeding value correlation between both EBVs was 0.99. Consequently, identical BS were selected in both scenarios across all constraints for genetic relationships. As expected from the curve pattern for genetic merit, the reduction in the number of selected BS was strongest when relaxing the genetic relationship constraint at already low levels for genetic relationships.

**FIGURE 3:**
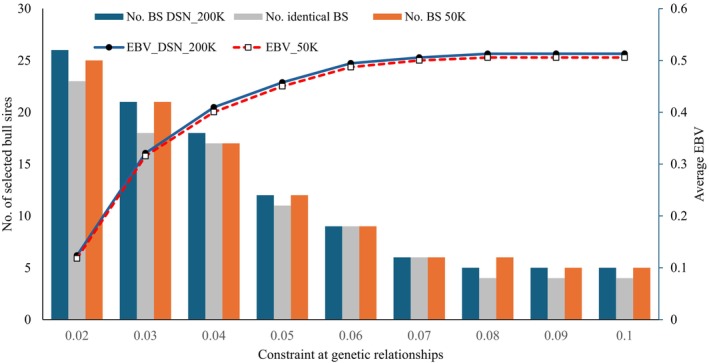
Average estimated breeding values (EBV) for the fat‐to‐protein ratio of selected bull sires (BS) and bull dams and the number of selected BS at constraint for average genetic relationships. (DSN_200 K = the DSN specific SNP chip was used for the genetic evaluation; 50 K = the standard 50 K chip was used for the genetic evaluations). [Colour figure can be viewed at wileyonlinelibrary.com]

Figure 3 displays genetic merit and the number of selected BS at different constraint for genetic relationships for the OGC selection for FPR. The shape of curves for genetic merit and number of selected BS follows the pattern as described for Pro% and STAT. A more relaxed genetic relationship constraint was associated with favourable effects on the average EBV for FPR, contributing to prevent both diseases acidosis and ketosis. In contrast to STAT, the selected BS were not identical based on 50 K EBV or on DSN_200 K EBV, albeit the differences were quite small. With regard to the intermediate optimum trait FPR and the applied EBV transformation, there is no guarantee that for each BD with a value above the optimum there is an appropriate mating partner with a value below the optimum, and vice versa. Nevertheless, the small number of finally selected BD and BS for a genetic relationship constraint larger than 0.04 had favourable EBVs in the sense of metabolic stability. These BS and BD had original FPR EBV very close to zero, implying large transformed FPR EBVs. The large transformed FPR EBVs disprove any concerns for either ketosis or acidosis, ultimately contributing to stabilised selection for FPR.

**FIGURE 4:**
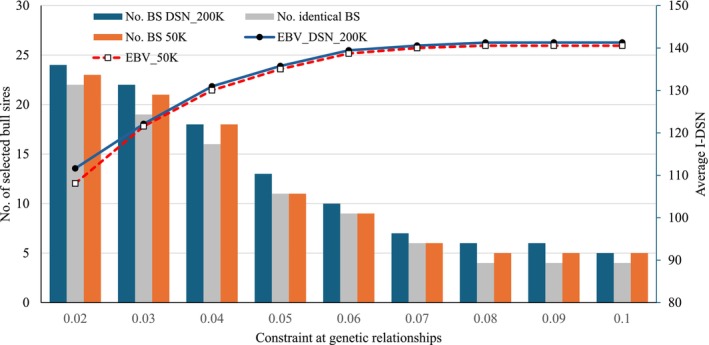
Average index (I‐DSN) of selected bull sires (BS) and bull dams and the number of selected BS at constraint for average genetic relationships. (DSN_200 K = the DSN specific SNP chip was used for the genetic evaluation; 50 K = the standard 50 K chip was used for the genetic evaluations). [Colour figure can be viewed at wileyonlinelibrary.com]

The OGC results for the selection based on I‐DSN are depicted in Figure 4, indicating the same curve pattern for the genetic merit and the number of selected BS as observed for single traits. Again, both EBV sources (DSN_200 K vs. 50 K) only had very minor effects on the selection of elite animals.

Overall, the OGC application is in agreement with the DSN breeding objectives, that is, to maintain a balance between long‐term genetic gain and genetic diversity in terms of genetic relationships. For all three traits Pro%, STAT and FPR, as well as for I‐DSN, a genetic relationship constraint larger than 0.06 was associated with only minor additional genetic merit. Consequently, the 0.06 genetic relationship constraint is suggested for OGC applications in the current DSN breeding program. The average pedigree relationship coefficient among the elite animals used for matings in practice (5 selected BS and 30 selected BD) is 0.058, which yielded average EBVs of 0.25 for Pro%, of −4.10 for STAT and of 0.42 for FPR. The respective average EBVs of selected BS and BD based on OGC (50 K EBV) were 0.31 for Pro%, −2.96 for STAT and 0.49 for FPR, indicating additional genetic merit in the range from 17% to 27% for the different traits compared to the reference scenario (current DSN selection scheme as practiced by the breeding organisation). With regard to the constructed index, OGC selection at a 0.06 constraint for genetic relationships resulted in a genetic merit of 139.4 (DSN_200K). The average I‐DSN of BD and BS selected by the breeding organisation was 118.1, indicating a genetic gain of 17.9%.

## 4 Discussion

### 4.1 Single‐Step Genetic Parameters and Breeding Values

Utilisation of the DSN specific SNP chip (DSN_200 K) or of the commercial 50 K resulted in almost identical results in single‐step genetic parameter estimations for variance components and heritabilities for all three traits Pro%, STAT and FPR. In genomic evaluations for production traits in DSN, Wolf et al. (2023) modelled pure genomic relationship matrices (G‐matrices) considering different marker densities. Similarly to our study, they found that genetic parameters based on the specific DSN_200 K or on whole‐genome sequence data did not differ significantly from the results based on the 50 K or a randomly constructed 200 SNP chip. As outlined in previous genomic evaluations with G‐matrices, the chosen genotype platform or even utilisation of whole‐genome sequences, had minor impact on genetic parameter estimates (e.g., van Binsbergen et al. 2015). Instead, the size and the genetic composition of the training set was the crucial parameter influencing the genetic parameter estimates (e.g., Naderi, Yin, and König 2016). In contrast to genomic evaluations only considering the G‐matrix, pedigree relationships additionally determine the outcome in single‐step approaches, and pedigree relationships are completely independent from the marker panel or the marker density. Hence, the even higher similarity of genetic parameter estimates from different SNP chips in the present single‐step study compared to “G‐approaches” could be expected. The correlation coefficients larger than 0.99 between EBVs from the DSN_200 K and the 50 K in same traits are a further sign in this regard. A breed specific SNP chip might have greater application potential in breeds with larger genetic distances to HF than the DSN. The still existing genetic relationship between DSN and HF (Naderi et al. 2020) might explain the only minor differences in the present study.

In the present study, all variance components and heritabilities had quite small standard errors. Major reasons for the reliable genetic parameter estimates and EBVs are the large datasets of phenotyped cows for all three traits Pro%, FPR and STAT, and the high quality of the pedigree relationships, because several gaps in the pedigree could be filled by applying the DSN specific pedigree imputation algorithm (Jaeger, Brügemann, Naderi, Brandt, and König, 2018; Jaeger, Scheper, König, and Brügemann, 2018). The importance of the high quantity and quality of phenotyped cows in genomic predictions based on so‐called cow training sets was especially highlighted for local breeds, which are characterised by a small number of AI sires with highly accurate conventional EBV (Schöpke and Swalve 2016). The heritabilities in the DSN population for all three traits are at the upper range compared to estimates in commercial breeds with large population size, especially for STAT. An explanation might be the accurate and objective STAT measurements in cm in the current study compared to subjective classifier scores (e.g., Schierenbeck, König, and Simianer 2009). Similarly to our study, large heritabilities for Pro% have been reported in other cattle populations, and a large heritability especially for DSN was expected, because of the identified major candidate genes with large effects (Korkuć et al. 2023).

The genetic‐statistical modelling approach, that is, with or without regressions on the pedigree‐based inbreeding coefficient, had no effect on the genetic parameter estimates. Similarly, in HF cows from Iran, Rokouei et al. (2011) applied genetic‐statistical models including the pedigree relationship matrix, and additionally considering or ignoring a linear regression on the pedigree‐based inbreeding coefficients. Variance components and genetic parameters for production and reproduction traits from both models were very similar, and the rank correlations between EBVs for male animals were throughout larger than 0.95. Fioretti et al. (2001) enhanced genetic models by additionally considering the inbreeding effect of the sire and the dam as linear regressions, but the maximum change in heritabilities compared to the baseline model was only 0.01.

### 4.2 Inbreeding Effects

In the present study, the negligible effects of inbreeding on genetic parameter estimates might be due the generally small effects of inbreeding on the cow traits Pro%, STAT and FPR. In Australian HF, Pryce et al. (2014) indicated generally stronger inbreeding depressions in genomic models compared to pedigree‐based analyses. However, the negligible detrimental effects of inbreeding on production traits in the present DSN study are in agreement with results from pure genomic approaches in Canadian and Dutch HF populations (Makanjuola et al. 2020; Doekes et al. 2019, respectively). In their studies, genomic inbreeding measures such as runs of homozygosity or coefficients from the genomic relationship matrix, outlined inbreeding depressions only for a very few low heritability functional traits. Results from simulation studies indicated over‐estimated inbreeding depressions based on inbreeding coefficients from the genomic relationship matrix in populations with small effective population size (Caballero, Villanueva, and Druet 2020).

### 4.3 Optimum Genetic Contribution Selection

The current pedigree inbreeding coefficient for DSN bulls and cows from the most recent birth year of 0.038 is on a generally moderate level compared to other cattle populations (e.g., Hinrichs et al. 2015). However, the quite close genetic relationships of 0.079 among the current available AI sires and the obvious increase of inbreeding in the most recent birth years suggest to implement a proper inbreeding management in DSN breeding approaches. The reason for the accumulation of inbreeding and genetic relationships in DSN in the recent years might be the shift from the strong utilisation of natural service sires towards AI. In this regard, Jaeger, Brügemann, Naderi, Brandt, and König (2018) recently identified close genetic relationships among intensively used AI sires, and they suggested to control genetic relationships in a long‐term perspective through the determination of optimum genetic contributions for BD and BS. Application of mating programs within cow herds, that is, to mate a specific cow sire with a specific cow dam, only supports to minimise inbreeding in a short‐term, but not in long‐term perspective (König and Simianer 2006).

Generally, the applied OGC approach in the DSN breed based on genomic breeding values from single‐step genomic evaluations are in agreement with previous pure pedigree‐based approaches, or with enhanced genomic applications. The general pattern of associations found in the present study, that is, a quite strong accumulation of genetic merit with a relaxing constraint at low genetic relationship levels, but only minor effects on EBVs at already high levels, have been displayed by Kearney et al. (2004) in the UK HF population, and by König and Simianer (2006) in German HF. Both latter studies used pedigree EBV and constraints for the pedigree relationships. For local breeds, Biermann et al. (2014) emphasised the importance of natural matings, implying difficulties to mate elite animals according to their suggested genetic contributions. Consequently, they enhanced the specific mating algorithm by Sonesson and Meuwissen (2000) through a second constraint, which considered the regional availability of sires. However, in DSN, the percentage of AI is larger than 80% and almost 100% in the BD herds. Additionally, an exchange of natural service sires has been observed across producer herds (Jaeger, Brügemann, Naderi, Brandt, and König, 2018; Jaeger, Scheper, König, and Brügemann, 2018).

In some local cattle breeds with small population size, crossbreeding with related breeds is common practice, for example, between Angler and dual‐purpose Red and White cattle (Addo et al. 2017). Consequently, to account for the breed origin of alleles, Wang, Bennewitz, and Wellmann (2017) additionally considered migration, genetic uniqueness and native allele diversity in optimum contribution selection for Vorderwald and Angler cattle. In this regard, for the handling of genetic introgression with other breeds, the software package Optisel (Wellmann 2019) can consider native contributions from pedigree data, or segment‐based kinships from genomic marker data. Nevertheless, such aspects might be important for some local cattle breeds with historical or recent migration, but not for DSN. DSN has a strong purebred history, implying that migration of other breeds was only practiced up to the end of the 19th century (Mügge et al. 1999). To enlarge the DSN gene pool, Jaeger, Scheper, König, and Brügemann (2018) suggested across‐country genetic evaluations considering the purebred DSN from Poland and The Netherlands. For such an international purebreed approach, the OGC concept from this study can be directly applied.

An increase of BS for elite matings (being a major result from the current OGC applications) is a very efficient tool to maintain long‐term genetic diversity in AI programs for dairy cattle. It was the widespread use of frozen semen of influential sires in the late 1960s and early 1970s, which mainly contributed to a fast increase of genetic relationships in German HF, with long‐term impact on the current population structure (König and Simianer 2006). Accordingly, in all three continents Europe, North America and Oceania, Miglior (2000) identified the same five influential BS contributing to more than 50% to the young bull generations. The 14 (for Pro%), the nine (for FPR), the seven (for STAT) and the 10 (for I‐DSN) selected BS in the present OGC approach at a 0.06 constraint for the genetic relationship exceeds the number of only five bull sires, which are currently used in practical DSN breeding schemes. In practice, the DSN breeding organisation suggests the same BS to improve the overall breeding goal, which is mainly determined by Pro%, FPR and STAT. In the current OGC approach, three BS were in common between all three traits and for I‐DSN. Brito et al. (2021) associated loss of diversity with intensive selection on only a few traits reflecting a specific trait category, or on a specific breeding index. König, Brügemann, and Pimentel (2013) draw similar conclusions, especially if the few breeding traits are genetically closely correlated. In contrast, the three traits Pro%, FPR and STAT reflect different trait categories with different underlying physiological and genetic mechanisms. However, from a practical perspective, it might be difficult to implement specific BS and BD selection strategies for different traits. Consequently, genetic improvements based on optimum genetic contributions for I‐DSN, is the most realistic approach.

## 5 Conclusion

Heritabilities for the major breeding goal traits from the single‐step genetic parameter estimations in the local DSN breed were quite large for Pro% (0.69) and STAT (0.78), but quite small for FPR (0.11). The choice of the SNP chip (50 K vs. DSN_200 K) as well as of the statistical modelling approach (with or without a linear regression on pedigree‐based inbreeding coefficients), implied negligible effects on all variance components. An increase of inbreeding was associated with only small unfavourable or neutral effects on Pro%, STAT and FPR. Optimum genetic contribution selection based on single‐step EBVs for BS and BD led to an increase in genetic merit and a decline in the number of selected BS with a relaxed constraint for the genetic relationship. However, the respective effects were marginal when relaxing the constraint for the genetic relationship at 0.06 or higher. Consequently, we suggest OGC applications with a constraint of genetic relationships at 0.06, which contributed to a genetic gain of 17.9% in the overall index I‐DSN and more diversity due to an increased number of BS when compared to the current practical DSN elite animal selection scheme.

## Conflicts of Interest

The authors declare no conflicts of interest.

## Data Availability

The data that support the findings of this study are available from the corresponding author upon reasonable request.
